# Increased A20-E3 ubiquitin ligase interactions in *bid*-deficient glia attenuate TLR3- and TLR4-induced inflammation

**DOI:** 10.1186/s12974-018-1143-3

**Published:** 2018-05-02

**Authors:** Sinéad Kinsella, Michael Fichtner, Orla Watters, Hans-Georg König, Jochen H. M. Prehn

**Affiliations:** 1 Department of Physiology and Medical Physics, Centre for the Study of Neurological Disorders, Royal College of Surgeons in Ireland, 123 St. Stephen’s Green, Dublin 2, Ireland; 2 Program in Immunology, Clinical Research Division, Fred Hutchinson Cancer Research Center, 1100 Fairview Ave N, Seattle, WA 98109 USA; 3 Department of Physiology and Medical Physics, Royal College of Surgeons in Ireland, 123 St. Stephen’s Green, Dublin 2, Ireland

**Keywords:** A20, Bid, E3 ubiquitin ligase, Toll-like receptor 4, Ubiquitination

## Abstract

**Background:**

Chronic pro-inflammatory signaling propagates damage to neural tissue and affects the rate of disease progression. Increased activation of Toll-like receptors (TLRs), master regulators of the innate immune response, is implicated in the etiology of several neuropathologies including amyotrophic lateral sclerosis, Alzheimer’s disease, and Parkinson’s disease. Previously, we identified that the Bcl-2 family protein BH3-interacting domain death agonist (Bid) potentiates the TLR4-NF-κB pro-inflammatory response in glia, and specifically characterized an interaction between Bid and TNF receptor associated factor 6 (TRAF6) in microglia in response to TLR4 activation.

**Methods:**

We assessed the activation of mitogen-activated protein kinase (MAPK) and interferon regulatory factor 3 (IRF3) inflammatory pathways in response to TLR3 and TLR4 agonists in wild-type (wt) and *bid*-deficient microglia and macrophages, using Western blot and qPCR, focusing on the response of the E3 ubiquitin ligases Pellino 1 (Peli1) and TRAF3 in the absence of microglial and astrocytic Bid. Additionally, by Western blot, we investigated the Bid-dependent turnover of Peli1 and TRAF3 in wt and *bid*^−/−^ microglia using the proteasome inhibitor Bortezomib. Interactions between the de-ubiquitinating Smad6-A20 and the E3 ubiquitin ligases, TRAF3 and TRAF6, were determined by FLAG pull-down in TRAF6-FLAG or Smad6-FLAG overexpressing wt and *bid*-deficient mixed glia.

**Results:**

We elucidated a positive role of Bid in both TIR-domain-containing adapter-inducing interferon-β (TRIF)- and myeloid differentiation primary response 88 (MyD88)-dependent pathways downstream of TLR4, concurrently implicating TLR3-induced inflammation. We identified that *Peli1* mRNA levels were significantly reduced in PolyI:C- and lipopolysaccharide (LPS)-stimulated *bid*-deficient microglia, suggesting disturbed IRF3 activation. Differential regulation of TRAF3 and Peli1, both essential E3 ubiquitin ligases facilitating TRIF-dependent signaling, was observed between wt and *bid*^*−/−*^ microglia and astrocytes. *bid* deficiency resulted in increased A20-E3 ubiquitin ligase protein interactions in glia, specifically A20-TRAF6 and A20-TRAF3, implicating enhanced de-ubiquitination as the mechanism of action by which E3 ligase activity is perturbed. Furthermore, Smad6-facilitated recruitment of the de-ubiquitinase A20 to E3-ligases occurred in a *bid*-dependent manner.

**Conclusions:**

This study demonstrates that Bid promotes E3 ubiquitin ligase-mediated signaling downstream of TLR3 and TLR4 and provides further evidence for the potential of Bid inhibition as a therapeutic for the attenuation of the robust pro-inflammatory response culminating in TLR activation.

**Electronic supplementary material:**

The online version of this article (10.1186/s12974-018-1143-3) contains supplementary material, which is available to authorized users.

## Background

Neuroinflammation is a contributing factor to the pathogenesis of multiple diseases of the central nervous system (CNS), including Alzheimer’s disease [1, 2], Amyotrophic Lateral Sclerosis [3, 4], Parkinson’s disease [5], stroke [6], and epilepsy [7]. Attenuating the robust pro-inflammatory response associated with the diseased brain is crucial for preventing neuronal death. Accumulation of activated microglia and astrocytes in areas of localized neurodegeneration has been reported [8], with chronic microglial polarization towards the pro-inflammatory M1 state demonstrated to induce neuronal death [9, 10] and initiate the infiltration of peripheral immune cells, such as macrophages [11].

A plethora of evidence exists for the involvement of TLRs in neuropathogenesis [12–18]. Glial crosstalk potentiates a pro-inflammatory feedback loop, with evidence for Toll-like receptor (TLR) signaling in the recruitment of the adaptive immune response [11]. TLRs are highly conserved master regulators of the cellular innate immune response [19, 20], responsible for the initiation and propagation of the inflammatory cascade in response to bacterial, viral or microbial pathogens, and nucleic acids, known as pathogen-associated molecular patterns (PAMPs) or danger-associated molecular pattern (DAMPs) [21–23].

TLR4-induced activation of multiple sophisticated protein complexes occurs via the MyD88- and TRIF-dependent pathways [24]. Ubiquitin, a 76 amino acid cellular protein which covalently attaches to a target molecule to form ubiquitin chains, facilitates the multifunctional post translational modification termed ubiquitination, resulting in enhanced turnover or altered signal transduction of the target protein [25–27]. Ubiquitination plays a critical role in the orchestration of the innate immune response [28], with downstream signaling from TLRs tightly regulated by a number of E3 ubiquitin ligases, such as Peli1, TRAF3, and TRAF6, which facilitate the polyubiquitination of several critical components mediating the pro-inflammatory response [27, 29, 30]. Peli1 is a positive regulator of both MyD88- and TRIF-dependent TLR4 activation, promoting the assembly of signaling complexes in a K63-ubiquitin-dependent manner, and is identified as a key player in both the MyD88-IRAK1-TAK1-TRAF6 and TRIF-TBK1 axes [31, 32]. TRAF3 has differential roles in the regulation in the MyD88- and TRIF-dependent pathway dictated by the alternative ubiquitination of TRAF3 [33]. TRAF6-mediated K63-linked ubiquitination of the IKK complex subunit, IKKγ, positively activates NF-κB signaling in a MyD88- [34] and TRIF-dependent manner [29, 35]. Additionally, ubiquitination is modulated by a number of de-ubiquitinating enzymes (DUBs), specifically A20 (also known as TNFAIP3), which negatively regulate the TLR-induced response by ensuring the cleavage and removal of polyubiquitin chains from target proteins and thus terminate signaling [36–38]. Dysregulated A20 expression and polymorphisms in *tnfaip3* are associated with multiple inflammatory diseases [39, 40], with spontaneous neuroinflammation reported in *A20*-deficient mice [41]. Furthermore, overexpression of A20 was shown to be protective in epilepsy [42] and cerebral ischemia [43].

BH3-interacting domain death agonist (Bid) is a pro-apoptotic BH3-only domain Bcl2 (B cell lymphoma 2) family member [44], and a key executioner of the intrinsic apoptotic pathway, linking death receptor activation to the mitochondrial apoptosis pathway [45]. Bid is cleaved by caspase-8 to its truncated form tBid, which subsequently leads to outer mitochondrial membrane permeabilization [46], initiating the caspase cascade [47]. Several studies have suggested additional non-apoptotic roles of Bid, including modulation of inflammatory signaling by associating with the IKK complex in astrocytes [48], and NOD2 receptors in intestinal epithelia [49]. Importantly, Bid was shown to modulate the immunological profile in both microglia and macrophages [50].

Previously, we have demonstrated that microglial Bid positively regulates TLR4 signaling by promoting the K63-linked polyubiquitination of TRAF6 [51]. Here, we further elucidated the mechanism of action of Bid-dependent polyubiquitination of several E3-ligases and identified increased associations between the de-ubiquitinase A20 and the E3 ligases TRAF3, TRAF6, and Peli1 in *bid*-deficient glia and macrophages. Collectively, these results demonstrate that Bid modulates MyD88- and TRIF-dependent signaling by regulating protein interactions and attenuating the cleavage of polyubiquitin chains, thereby facilitating downstream signaling and propagating a chronic pro-inflammatory response.

## Methods

### Antibodies and reagents

All common chemicals were obtained from Sigma-Aldrich (Wicklow, Ireland) unless otherwise stated. Antibodies used for Western blot and co-immunoprecipitation include rabbit anti-Bid (Abcam, ab62469, 1:1000), rabbit anti-Peli1 (Abcam ab199336, 1:1000), rabbit anti-TRAF3 (Abcam, ab76147, 1:500), mouse anti-TRAF6 (Santa Cruz, sc8409, 1:200), rabbit anti-phospho-IRF3 (Cell Signaling, 4947P, 1:500), rabbit anti-phospho-TBK1 (Cell Signaling, 5483P, 1:500), rabbit anti-phospho c-Jun (Cell Signaling, 9261S, 1:1000), rabbit anti-A20/TNFAIP3 (Cell Signaling, 5630, 1:1000), rabbit anti-LC3 (Abcam, ab51520, 1:2000), rabbit anti-FLAG (OctA-Probe D-8, Santa Cruz, sc-807, 1:500), anti- α-Tubulin (Sigma-Aldrich, T6199, 1:5000), anti-β-Actin (Sigma-Aldrich, A3853, 1:5000), and anti-GAPDH (Abcam, ab8245-100,1:5000). Bortezomib was obtained from Millennium Pharmaceuticals (Cambridge, MA, USA). Lipopolysaccharide (LPS) was obtained from (Sigma-Aldrich, L4391), and PolyI:C (31852-29-6) and Pam_3_CSK4 (112208-00-1) were obtained from InvivoGen.

### Preparation of astrocytes and microglia

Mixed glial cultures were obtained from the cortices of P0–P2 wt and *bid*^*−/−*^ mice of mixed sexes on a *C57BL/6* background, as described previously [51]. Briefly, the cortices were isolated, the meninges were removed, and the tissue was incubated with Trypsin-EDTA at 37 °C for 10 min. The Trypsin-EDTA was removed and replaced with DMEM-F12/L-glutamine (Gibco, Life Technologies) containing Penicillin-Streptomycin (1%, Sigma-Aldrich) and fetal bovine serum (10%, Sigma-Aldrich). The cells were triturated and passed through a 40-μm nylon cell strainer (BD Falcon) before being centrifuged at 300 × *g* for 5 min. The cells were plated at a density of 2 cortices/T75 flask and cultured for 10 days in the presence of M-CSF (10 ng/ml, R & D Systems) and GM-CSF (20 ng/ml, R & D Systems) in order to promote microglial proliferation [52]. Microglia were isolated from the co-culture, and the remaining cells were passaged twice and cultured in the absence of M-CSF and GM-CSF as astrocyte cultures. *Bid*^*−/−*^ mice were generated in the laboratory of Dr. Andreas Strasser, WEHI, Melbourne, Australia [53].

### Preparation of macrophages

Macrophages were obtained from the bone marrow of 6-week-old wt and *bid*^*−/−*^ mice of mixed sexes on a *C57BL/6* background. Briefly, the mouse was euthanized by cervical dislocation, and the femur and tibia were carefully removed. Following the complete removal of attached muscle, the bones were cut using a sterile scalpel in a sterile laminar flow hood, and the bone marrow was flushed out using a 27 G needle containing DMEM (Gibco, Life Technologies) supplemented with penicillin-streptomycin (1%, Sigma-Aldrich) and fetal bovine serum (10%, Sigma-Aldrich). The bone marrow was homogenized and passed through a 40-μm nylon cell strainer (BD Falcon) and centrifuged at 445 × *g* for 3 min. The cells were resuspended in red blood cell lysis buffer (8.26 g NH_4_Cl, 1 g KHCO_3_, 0.037 g EDTA) and incubated for 1 min at room temperature before addition of DMEM (plus penicillin-streptomycin and fetal bovine serum). The cells were centrifuged at 445 × *g* for 3 min and cultured for 10 days in DMEM plus Pen/Strep, plus FBS, containing 40 ng/ml M-CSF (10 ng/ml, R & D Systems), in order to stimulate macrophage proliferation.

### siRNA transfection

Macrophages were transfected (100 μM siRNA/3 × 10^5^ cells) with an siRNA targeting Bid, sequence ACACGACUGUCAACUUUAU, which was designed using an algorithm optimized for siRNA selection [54]. The macrophages were transfected using lipofectamine, and the optimal silencing of *bid* was determined by qPCR analysis to be 48 h post transfection. A control siRNA consisting of a scrambled nucleotide sequence was also used.

### Western blot

The astrocytes, microglia, or macrophages were stimulated with Pam_3_CSK4 (100 ng/ml), PolyI:C (100 ng/ml), or LPS (100 ng/ml), or Bortezomib (100 μM) in full serum media for the desired time point, and lysed in RIPA buffer, containing protease and phosphatase inhibitors (1:100). The cells were incubated on ice for 20 min, centrifuged at 14,000 rpm for 15 min, and the protein concentration was determined by BCA assay (Micro BCA protein determination kit, Thermo Scientific). Following the addition of 1 × Laemmli Buffer, the samples were boiled for 5 min and loaded onto 10, 12, or 15% polyacrylamide gels as appropriate. The transfer was carried out using semi-dry transfer apparatus and PVDF membrane for 1.5 h at 18 V, with the membranes exposed to Ponceau S and blocked in 3% milk for 1 h post transfer. The membranes were incubated with the primary antibodies in 3% milk either overnight at 4 °C or 2 h at room temperature, were washed in TBS-Tween-20 (0.05%), and were incubated in 3% blocking solution containing the appropriate secondary antibody (peroxidase-conjugated anti-mouse IgG, anti-rabbit IgG, or anti-goat IgG, Sigma, 1:5000, as appropriate) for 2 h at room temperature. The membrane was washed 3 times for 5 min in TBS-Tween20, exposed to ECL Chemilluminescent Reagent (Millipore) for 5 min before being imaged on a LAS-3000 Imager (Fuji, Sheffield, UK), and the quantification of protein levels were calculated using the optical density measurements from Western blot experiments and further normalized to respective loading control (α-tubulin, β-actin, or GAPDH).

### qPCR analysis

RNA was extracted from each sample using the Qiagen RNAeasy kit, and 0.5 μg RNA was used for cDNA synthesis, using random primers and Superscript RT-II (Invitrogen). Two microliters of each cDNA sample and 18 μl of Mastermix (1 μl 10 μM primer (forward and reverse), 10 μl SYBRgreen PCR Mix, 7 μl RNase-free H_2_0) were added to give a total volume of 20 μl per capillary tube. The following cycle parameters were applied; 95 °C for 15 min, 94 °C for 15 s, 57 °C for 25 s, and 72 °C for 30 s. *gapdh* was used as an internal control for each sample analyzed. Primers were designed using Primer3 (http://biotools.umassmed.edu/bioapps/primer3_www.cgi), and are between 150 and 250 base pairs, optimized for SYBR detection, and obtained from Sigma-Aldrich. The following sequences were used: *gapdh (mouse)* forward 5’ AACTTTGGCATTGTGGAAGG 3’, reverse 5’ ACACATTGGGGGTAGGAACA 3’; *Peli1* forward 5’ TGCCGAAATCAATCAATCAA 3’, reverse 5’ CAATGGAGTGTCACTGGGTG 3’. qPCR analysis was carried out on a Roche Lightcycler 2.0 using SYBRgreen (Quantitect SYBRgreen kit, Qiagen).

### Co-immunoprecipitation

Overexpression of TRAF6-FLAG, Smad6-FLAG, or Ubiquitin-HA was carried out by transfection of 2.5 μg plasmid/9 × 10^5^ cells in a T75 flask, using lipofectamine. Twenty hours post transfection, the cells were stimulated with Pam_3_CSK4 (100 ng/ml), PolyI:C (100 ng/ml), or LPS (100 ng/ml) for 1 h before lysis in RIPA buffer (Tris 50 mM, NaCl 150 mM, SDS 0.1%, Sodium-deoxycholate 0.5%, Triton X-100 or NP-40 1%, plus 1:100 Protease Inhibitor, Sigma). Co-immunoprecipitation or pull-down experiments were carried out using DynaBeads Protein G [35 μl of Dynabeads®/sample (100–250 μg protein), Life Technologies, 10007D] and a magnetic rack (Life Technologies). Briefly, the beads were washed in RIPA buffer and incubated with 5 μg primary antibody (in 200 μl PBS for 1 h rotating at room temperature) and washed 3 times for 5 min in PBS buffer before equal amounts of protein (100–250 μg) were incubated for 2 h at room temperature (in a total volume of 750 μl). Elution of the samples from the beads using the magnetic rack, where the protein was denatured in RIPA buffer plus 1 × Laemmli buffer by incubating for 10 min at 70 °C, was followed by gel electrophoresis.

### Statistical analysis

Statistical analysis was carried out using GraphPad Prism software (GraphPad Software Inc., La Jolla, CA, USA), and the results are represented as mean ± SD. Statistical significance and *p* values were determined using the tests as detailed in the figure legends (* denotes *p* ≤ 0.05).

## Results

### Bid-dependent activation of IRF3 and JNK pathways in response to TLR3 and TLR4 stimulation

**Fig. 1:**
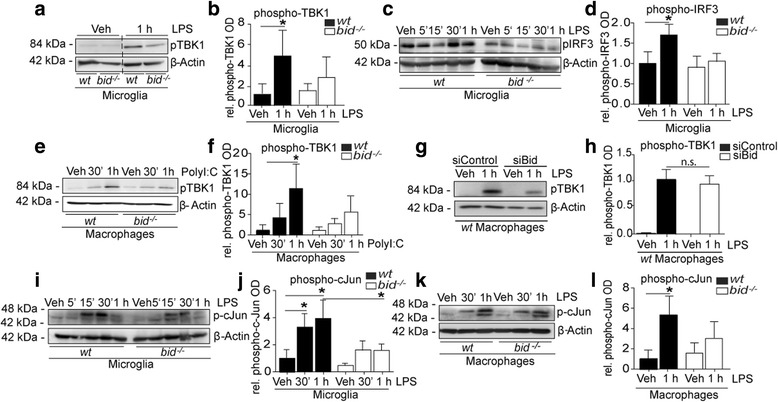
Reduced phosphorylation of TBK1, IRF3, and c-Jun in bid-deficient glia and macrophages compared with wt. **a**, **b** wt and *bid*-deficient microglia were stimulated with LPS (100 ng/ml) for 1 h and lysed in RIPA buffer, and phosphorylated TBK1 levels were determined by Western blot (*n* = 6–7, 6–7 cultures from 6 separate platings, *p* = 0.0068, Kruskall-Wallis, Dunn’s non-parametric multiple comparison post hoc test). **c**, **d** wt and *bid*-deficient microglia were stimulated with LPS (100 ng/ml) for 5, 15, and 30 min or 1 h and lysed in RIPA buffer, and phosphorylated-IRF3 levels were determined by Western blot (*n* = 3, 3 cultures from 3 separate platings, *p* = 0.041, paired two-tailed *t* test, wt Veh vs. LPS 1 h). **e**, **f** wt and *bid*^*−/−*^ macrophages were stimulated with PolyI:C (100 ng/ml) for 30 min or 1 h and lysed in RIPA buffer, and phosphorylated-TBK1 levels were determined by Western blot (*n* = 4–6, 4–6 cultures from 5 separate platings, *p* = 0.0006 one-way ANOVA, Tukey’s multiple comparison post hoc test). **g**, **h** wt macrophages were transfected with an siRNA targeting Bid. Forty-eight hours post transfection, when Bid is optimally silenced, the cells were stimulated with LPS (100 ng/ml) for 1 h and lysed in RIPA buffer, and phosphorylated-TBK1 levels were determined by Western blot (*n* = 3, 3 cultures from 2 separate platings, *p* = 0.0588, paired two-tailed *t* test siControl LPS 1 h vs. siBid LPS 1 h). **i**, **j** wt and *bid*-deficient microglia were stimulated with LPS (100 ng/ml) for 5, 15, and 30 min or 1 h and lysed in RIPA buffer, and phosphorylated c-Jun levels were determined by Western blot (*n* = 5–7, 5–7 cultures from 6 separate platings, *p* < 0.0001, one-way ANOVA, Tukey’s multiple comparison post hoc test). **k**, **l** wt and *bid*-deficient macrophages were stimulated with LPS (100 ng/ml) for 30 min or 1 h and lysed in RIPA buffer, and phosphorylated c-Jun levels were determined by Western blot (*n* = 5–7, 5–7 cultures from 5 separate platings, *p* = 0.0017 one-way ANOVA, Tukey’s multiple comparison post hoc test). All samples were lysed in RIPA buffer containing protease and phosphatase inhibitors. The protein levels were quantified using optical densities. The data are represented as mean ± SD

Previously, we have demonstrated that *bid* deficiency in astrocytes [48] and microglia [51] results in reduced TLR4-induced NF-κB activation, which was identified to occur via Bid-induced TRAF6 polyubiquitination. TRAF6 mediates NF-κB signaling [55] and additionally facilitates the activation of mitogen-activated protein kinases (MAPK) and interferon regulatory factor 3 (IRF3) [56]. TRIF-dependent signaling requires the phosphorylation of TANK-binding kinase 1 (TBK1) and the subsequent association with the IKK subunit IҡB kinase ε (IKKε) to activate the transcription factor IRF3 [57]. Moreover, transforming growth factor β-activated kinase 1 (TAK1)-mediated phosphorylation of Jun N-terminal kinase (JNK) is proposed to induce IRF3 activation, thus suggesting that crosstalk between the MyD88-independent and -dependent pathways with respect to IRF3 activation is mediated via JNK activation [58]. Here, we further investigated the Bid-dependent effects on the regulation of TLR4 downstream signaling, focusing on IRF3 activation and JNK-mediated MAPK activation. We observed reduced phosphorylation of TBK1 (Fig. 1a, b, 4.59 fold ± 3.6 increase vs. 2.85 fold ± 3.0 increase ± SD; Fig. 1e, f, 11.4 fold ± 7.3 increase vs. 5.6 fold ± 4.8 increase ± SD), IRF3 (Fig. 1c, d, 1.7 fold ± 0.73 increase vs. 1.0 fold ± 0.65 increase ± SD), and c-Jun (Fig. 1i, j, 3.9 fold ± 2.8 increase vs. 1.5 fold ± 1.4 increase ± SD; Fig. 1k, l, 5.3 fold ± 3.5 increase vs. 3.0 fold ± 3.0 increase ± SD) in *bid*^*−/−*^ microglia and macrophages compared with their wt counterparts. Additionally, silencing of *bid* using an siRNA showed a tendency towards lower levels of TLR4-induced phosphorylation of TBK1 in wt macrophages compared with scrambled siRNA control-transfected macrophages (Fig. 1g, h, 0.71 ± 0.29 fold decrease ± SD, Con LPS vs. siBid LPS).

### Differential regulation of TRAF3 and Peli1 in TLR4-induced microglia, astrocytes, and macrophages

The critical role of the E3 ubiquitin ligase Peli1 in TLR signaling [59, 60] and in microglial-mediated CNS inflammation has been highlighted previously [61]. Peli1 facilitates TRAF6-mediated downstream signaling to MAPK [61]. Additionally, Peli1-mediated K63-linked polyubquitination of TBK1 is induced upon LPS stimulation activating IRF3 [32], with subsequent bidirectional signaling leading to the TBK1/IKKε-induced phosphorylation of Peli1 [62].

MyD88-dependent signal transduction to TAK1 requires the dissociation of the TRAF6-Peli1-IRAK complex from the membrane-bound receptor complex [33]. It is proposed that the cytoplasmic translocation of this complex, regulated by Peli1-mediated degradation of TRAF3, is essential for MAPK activation [33, 61]. In this way, TRAF3 negatively regulates MyD88 signaling, with the induction of pro-inflammatory genes restored in *Peli1*-deficient mice by the depletion of TRAF3 [61] and positively regulates the TRIF-dependent pathway mediating IRF3 activation, by executing K63-linked autoubiquitination on interaction with TRIF, which does not require cIAP1 or cIAP2 [33].

**Fig. 2:**
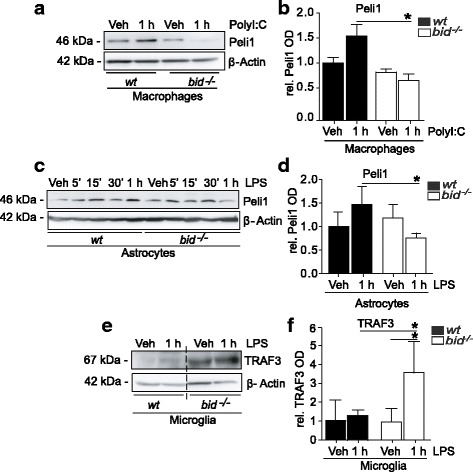
Differential induction of Peli1 and TRAF3 degradation in TLR3- and TLR4-stimulated bid-deficient glia. **a**, **b** wt and *bid*^*−/−*^ macrophages were stimulated with PolyI:C (100 ng/ml) for 1 h, lysed in RIPA buffer, and prepared for Western blot where Peli1 levels were determined (*n* = 5, 5 cultures from 4 separate platings, *p* = 0.004 one-way ANOVA, Tukey’s post hoc test). **c**, **d** wt and *bid*-deficient astrocytes were stimulated with LPS (100 ng/ml) for 5, 15, and 30 min and 1 h before being lysed in RIPA buffer and prepared for Western blot analysis of Peli1 levels (*n* = 4–5, 4–5 cultures from 3 separate platings, *p* = 0.031, Kruskall-Wallis, Dunn’s non-parametric multiple comparison post hoc test). **e**, **f** wt and *bid*-deficient microglia were stimulated with LPS (100 ng/ml) for 1 h and lysed in RIPA buffer, and TRAF3 levels were determined by Western blot (*n* = 3–4, 3–4 cultures from 4 separate platings, *p* = 0.019 one-way ANOVA, Tukey’s multiple comparison post hoc test). All samples were lysed in RIPA buffer containing protease and phosphatase inhibitors. The protein levels were quantified using optical densities. The data are represented as mean ± SD

Previously, we have identified a differential TLR4-induced response in Peli1 levels in wt and *bid*-deficient microglia [51]. We therefore also examined the levels of Peli1 upon both TLR3 and TLR4 activation and observed a lack of Peli1 induction in *bid*^*−/−*^ macrophages (Fig. 2a, b, 1.54 fold ± 0.22 increase vs. 0.65 fold ± 0.12 decrease ± SD) and astrocytes (Fig. 2c, d, 1.47 fold ± 0.9 increase vs. 0.75 fold ± 0.5 decrease ± SD). Additionally, increased TRAF3 levels were identified in *bid*^*−/−*^ microglia (Fig. 2e, f, 1.65 fold ± 1.3 increase vs. 3.5 fold ± 1.7 increase ± SD).

### Bid promotes *Peli1* transcription in response to TLR3 and TLR4 activation

**Fig. 3:**
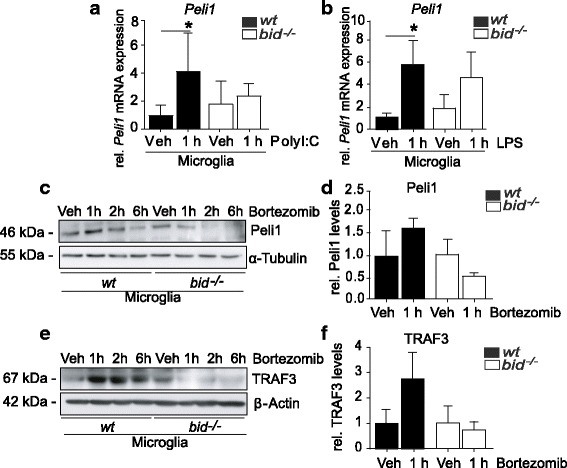
Bid-dependent induction of Peli1 in TLR3- and TLR4-activated microglia*.* **a** wt and *bid*-deficient microglia were stimulated with PolyI:C (100 ng/ml) for 1 h, lysed in RLT buffer, and prepared for qPCR analysis where *Peli1 mRNA* was quantified using the LightCycler (*n* = 6–7, 6–7 cultures from 6 separate platings, *p* = 0.0143, Kruskall-Wallis, Dunn’s non-parametric multiple comparison post hoc test)**. b** wt and *bid*-deficient microglia were stimulated with LPS (100 ng/ml) for 1 h, lysed in RLT buffer, and prepared for qPCR analysis where *Peli1 mRNA* was quantified using the LightCycler (*n* = 5–7, 5–7 cultures from 3 separate platings, *p* = 0.0025 one-way ANOVA, Tukey’s multiple comparison post hoc test). **c**, **d** wt and *bid*-deficient microglia were treated with Bortezomib (100 μM) for 1, 2, or 6 h, before being lysed in RIPA buffer, and Peli1 levels were determined by Western blot (*n* = 3–4, 3–4 cultures from 4 separate platings, *p* = 0.072, one-way ANOVA). **e**, **f** wt and *bid*-deficient microglia were treated with Bortezomib (100 μM) for 1, 2, or 6 h, before being lysed in RIPA buffer, and TRAF3 levels were determined by Western blot (*n* = 3, 3 cultures from 3 separate platings, *p* = 0.123, one-way ANOVA). All samples were lysed in RIPA buffer containing protease and phosphatase inhibitors. The protein levels were quantified using optical densities. The data are represented as mean ± SD

Peli1 is a target gene of IRF3 [62]. We next investigated *Peli1* mRNA levels by qPCR and observed that *Peli1* transcription was reduced in *bid*-deficient microglia, compared with wt, following TLR3 activation (Fig. 3a, 4.2 fold ± 3.3 vs. 2.3 fold ± 1.5 increase ± SD) or TLR4 activation (Fig. 3b, 5.4 fold ± 2.6 increase vs. 4.3 fold ± 2.5 increase ± SD), suggesting Bid-dependent activation of the TRIF-IRF3 signaling pathway.

Additionally, we assessed the levels of Peli1 and TRAF3 upon inhibition of the proteasome in wt and *bid*-deficient microglia. Accumulation of TRAF3 in *Peli1*-deficient mice was identified to contribute to inflammatory pathogenesis in experimental autoimmune encephalitis (EAE) [31]. Peli1 is phosphorylated, ubiquitinated, and sumoylated [63, 64], and although Peli1 is degraded via the proteasome, it is unclear to date what targets Peli1 for degradation. Interestingly, when we examined the post translational effects of Bid, we demonstrated a tendency towards differential regulation of both Peli1 and TRAF3 in *bid*-deficient microglia, with a lack of accumulation of Peli1 (Fig. 3c, d, 1.65 fold ± 0.8 increase vs. 0.53 fold ± 0.71 fold decrease ± SD), and TRAF3 (Fig. 3e, f, 2.73 fold ± 1.5 increase vs. 0.74 fold ± 0.82 decrease ± SD) following 1 h Bortezomib treatment, suggesting a Bid-dependent regulation of E3 ligase stability.

### Increased associations of A20 with TRAF3 and TRAF6 in *bid*-deficient glia and macrophages are facilitated by Smad6-A20 complex recruitment

**Fig. 4:**
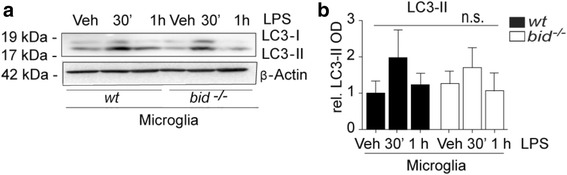
Bid deficiency does not alter autophagosome formation*.* **a**, **b** wt and *bid*-deficient microglia were stimulated with LPS (100 ng/ml) for 30 min or 1 h and lysed in RIPA buffer, and anti-LC3-II levels were determined by Western blot (*n* = 3, cultures from 3 separate platings, one-way ANOVA, *p* = 0.43). The data are represented as mean ± SD

Following the identification of a Bid-dependent dysregulated proteasomal degradation of Peli1 and TRAF3, we subsequently focused on the deubiquitinase A20. Inflammatory regulation in response to TLR4 activation is mediated by A20 which restricts signaling cascades by cleaving K48- and K64-linked polyubiquitin chains [38], thereby providing a negative feedback loop. A20 regulates TLR-induced autophagy by cleaving K63 polyubiquitin chains on Beclin 1, opposing the action of TRAF6 and limiting autophagy [65]. Therefore, we examined the role of Bid in autophagosome formation. However, we failed to observe differential regulation of LC3 lipidation in *bid*-deficient microglia stimulated with LPS, suggesting that Bid does not directly affect autophagy induction (Fig. 4a, b, 1.97 ± 1.18 vs. 1.7 ± 1.02 fold change ± SD).

**Fig. 5:**
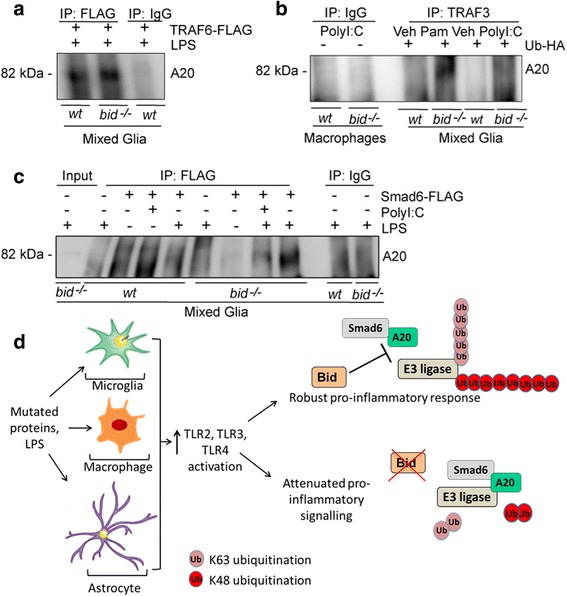
Increased A20-TRAF6, A20-TRAF3, and Smad6-A20 interactions in bid-deficient glia. **a** wt and *bid*^*−/−*^ mixed glia were transfected with TRAF6-FLAG and stimulated for 1 h with LPS (100 ng/ml) 20 h post transfection. The cells were lysed in RIPA buffer, and anti-FLAG was immunoprecipitated from the samples. A20-TRAF6-FLAG interaction was detected by Western blotting using an anti-A20 antibody (*n* = 2 separate experiments, repeated with similar results). **b** wt and *bid*^*−/−*^ glia were transfected with ubiquitin-HA for 20 h and subsequently stimulated with Pam_3_CSK4 (Pam, 100 ng/ml) or PolyI:C (100 ng/ml) for 1 h before being lysed in RIPA buffer. Anti-TRAF3 was co-immunoprecipitated from each of the samples, and TRAF3-A20 interactions were determined by Western blotting using anti-A20 (*n* = 1 experiment). **c** wt and *bid*-deficient mixed glia were transfected with Smad6-FLAG, or control vector pcDNA4.1, 20 h prior to stimulation with PolyI:C (100 ng/ml) or LPS (100 ng/ml) in full serum media. Following a 1-h stimulation, the cells were lysed in RIPA buffer, containing protease and phosphatase inhibitors, and the protein concentration was assessed using BCA assay. Anti-FLAG was immunoprecipitated from each of the samples, and the samples were prepared for Western blot analysis. The membrane was exposed to anti-A20 to determine the Smad6-FLAG-A20 interactions (*n* = 2 experiments, repeated with similar results).  All samples were lysed in RIPA buffer containing protease inhibitors, and protein concentrations were determined by BCA assay. Anti-IgG was used as co-immunoprecipitation and pull-down controls for each experiment. **d** Schematic showing the mechanism of action of Bid in response to TLR2 and − 3 and − 4 stimulation in glia and macrophages. Bid sequesters Smad6 and A20 thereby preventing Smad6-mediated recruitment of A20 to the E3 ligases, resulting in a lack of polyubiquitin chain cleavage and promotes pro-inflammatory signaling

Ubiquitin linkage to the target protein is a highly precise process, with the specificity of the substrate ensured by the E3 ubiquitin ligase [66], and each lysine residue (K6, K11, K27, K29, K33, K48, or K63) eliciting different linkage types leading to distinct functional outcomes [28]. TRAF6 undergoes K63-linked autoubiquitination and target-induced polyubiquitination [29, 34], whereas both TRAF3 [33, 61] and Peli1 [67, 68] accept K48- and K63-linked ubiquitin chains. A20 has been shown to cleave both K48- and K63-linked ubiquitin chains [38]. We next determined the interactions between A20 and the E3 ligases TRAF3 and TRAF6, determined by pull-down and co-immunoprecipitation assays, in the presence and absence of Bid, in order to determine a Bid-dependent effect on A20 interactions following TLR3 or TLR4 activation. A20-TRAF6 interactions were increased in LPS-stimulated *bid-*deficient glia upon TRAF6-FLAG overexpression (Fig. 5a), with a similar pattern observed in ubiquitin-HA (Ub-HA) overexpressing *bid-*deficient glia at rest and upon LPS activation (Additional file 1: Figure S1A). Additionally, TRAF6-Peli1 interactions were elevated in TRAF6-FLAG overexpressing LPS-induced wt glia, which was not observed in Ub-HA overexpressing *bid-*deficient glia at rest and upon LPS activation (Additional file 1: Figure S1B). Similarly, A20-TRAF3 associations were increased in Ub-HA overexpressing *bid-*deficient glia stimulated with the TLR2 agonist Pam3CSK4 (Pam), but not in Poly-I:C stimulated samples (Fig. 5b). Additionally, we observed increased A20-Peli1 interactions in *bid*-deficient macrophages upon LPS stimulation (Additional file 1: Figure S1C).

Smad6 mediates the recruitment of A20 to E3 ubiquitin ligases in response to TLR activation, allowing for the subsequent negative feedback in the form of polyubiquitin chain cleavage [69]. Smad6-A20 interactions were examined by FLAG pull-down in Smad6-FLAG overexpressing wt and *bid*-deficient mixed glia simulated with PolyI:C and LPS. The association between A20 and Smad6 was increased in both PolyI:C and LPS-stimulated *bid*-deficient compared with wt controls (Fig. 5c).

## Discussion

Ubiquitination plays a pivotal role in a plethora of signaling pathways, including apoptotic and inflammatory processes, with dysregulated ubiquitin activity described in multiple diseases (reviewed in [70]). Here, we show that the absence of *bid* in glia and macrophages results in increased interactions between A20 and the E3 ubiquitin ligases TRAF3 and TRAF6 in a Smad6-dependent manner, facilitating the cleavage of K48 and K63-linked ubiquitin chains, thereby inhibiting downstream signaling to NF-κB, MAPK, and IRF3, upon TLR3 and TLR4 activation.

TRAF6 was previously shown to promote the TAK1- and JNK-mediated phosphorylation of TBK1 and subsequently IRF3 activation [58]. We propose that the absence of Bid leads to constrained TRAF3 K48-linked degradation in microglia resulting in the attenuation of TRAF6 K63-linked signaling to TAK1 and therefore reduced phosphorylation of JNK. In addition, TRAF6 interacts with IRAK and Peli1 to form a complex that becomes dissociated from MyD88 and the TLR4 receptor, translocating to the cytoplasm where further downstream signaling occurs [33, 59]. Peli1 mediates TRAF6 K63-linked polyubiquitination of cIAP2 which induces the degradation of TRAF3 via the K48-linked ubiquitination of TRAF3 upon MyD88 signal induction [61]. Our data also demonstrates reduced phosphorylation of TBK1 in *bid*-deficient glia accompanied by reduced *Peli1* transcription, suggesting IRF3 activation is compromised by diminished MyD88 and TRIF pathway crosstalk. Peli1 mediates TRAF6- and TAK1 K63-linked polyubquitination in macrophages [32, 59]. Furthermore, Peli1 promotes the phosphorylation of TBK1 resulting in the enhanced activation of Peli1 [32, 62]. We propose that Bid-dependent regulation of Peli1 levels upon LPS stimulation may affect the interaction of Peli1 with the TBK1-IKKε (IκB kinase ε) complex, thus adding to the complexity to the role of Bid in TLR activation. Furthermore, we demonstrate that the Bid-dependent positive regulation of TLR4 signaling is not restricted to NF-κB, but also spans IRF3 activation and JNK-mediated MAPK activation, as demonstrated by the reduced levels of phosphorylated TBK1 in *bid*-deficient microglia and macrophages.

We propose the Bid-dependent attenuation of A20-mediated polyubiquitin chain cleavage accounts for the differential regulation of Peli1 and TRAF3 following LPS stimulation. A20, a ubiquitously expressed cytoplasmic protein, is a potent negative regulator of ubiquitin-dependent signaling, with perinatal lethality of *A20*-deficient mice accompanied by multi-organ inflammation [71]. Characterization of A20 identified ubiquitin ligase domains [38]. In addition, A20 has been shown to cleave unanchored K48-linked polyubiquitin chains [72], further adding to its mechanism of preventing degradation of proteins such as TRAF3.

A20 regulates LPS-induced autophagy by cleaving K63-linked polyubiquitin chains on Beclin-1 [73]. However, our data shows that the formation of LPS-induced autophagosomes is unaffected by *bid* deficiency in microglia, suggested a specificity of Bid-dependent A20 activity which may be cell type specific or limited to inflammatory signaling. Furthermore, as evidenced by the response of *bid*-deficient microglia to Bortezomib treatment, the absence of Bid may lead to sensitization towards autophagic rather than proteasomal degradation upon proteasomal inhibition.

Here, we have demonstrated that *bid*-deficient glia and macrophages have increased A20-E3 ligase interactions and increased Smad6-A20 interactions, specifically TLR4-induced TRAF6-A20 associations and Pam_2_CSK4-induced TRAF3-A20 associations. Additionally, reduced Peli1-TRAF6 interactions were observed in TLR4-stimulated *bid*-deficient glia, further implicating Bid-dependent modulation of ubiquitin ligase complexes that facilitate TLR-induced inflammatory signaling. Moreover, we previously demonstrated that Bid associates with TRAF6 in both unstimulated and LPS-stimulated conditions [51], and this study strengthens our previous finding by demonstrating A20-TRAF6 interactions are increased in both basal and LPS-activated *bid*-deficient glia.

We propose that Bid primarily regulates A20-E3 ligase and A20-Smad6 interactions in a MyD88-dependent manner, exerting its effects on TRIF signaling through TRIF-MyD88 crosstalk facilitated by phosphorylated JNK and the TBK1 complex. Delayed kinetics of the LPS-induced TRIF-dependent pathway has been described, highlighting dependence on the MyD88 pathway [74–76]. Furthermore, our data shows that Bid impairs Smad6-A20 complex formation, suggesting increased degradation of polyubiquitin chains in the MyD88 pathway in the absence of Bid. This is of key importance, not only in regulating innate immune signaling, but also in both the initiation of the adaptive immune response [77, 78], and TLR-mediated immune regulation in CD4+ T cells [79, 80] and dendritic cells [81].

In addition to the deubiquitinase activity of A20, interactions with E1 and E2 ubiquitin -activating enzymes demonstrate the central role of A20 in ubiquitin regulation [38]. Interestingly, associations between E2 and E3 ligases are disrupted by A20, specifically due to interactions between A20 and the E2 ubiquitin ligase Ubc13 [82]. Ubc13 is required for the formation of K63-linked polyubiquitin chains [83, 84], and *Ubc13-*deficient mice display a blunted LPS-induced NF-κB activation [85].

The role of Bid-dependent regulation of deubiquitination in TLR activation may also encompass NOD receptor signaling, as A20 facilitates the deubiquitination of proteins in both pathways [86], further elucidating previous studies implicating Bid and IKK complex association [49].

Targeting ubiquitin signaling is a promising mechanism to negatively regulate chronic inflammation in neurodegeneration and cancer. Therapeutics targeting ubiquitin include the NF-κB inhibitor, BAY 11-7802, which was recently been shown to interact with E2 ubiquitin ligases upon TLR4 activation, thus preventing K63-linked ubiquitin chain formation [87]. As BAY 11-7802 has multiple targets [88], a more specific ubiquitin-editing mechanism would be beneficial as an anti-inflammatory therapeutic. A20 is a highly potential target for ubiquitin-modulation, with astrocytic A20 shown to ameliorate EAE in mice [89]. Moreover, targeting A20 has potential for cancer therapeutics; however, disease specificity has been reported due to the pleiotropic nature of A20. Recently, a study demonstrated potent anti-inflammatory responses using Smad6 peptides, specifically by targeting Peli1 at the membrane-bound complex, thereby inhibiting downstream pro-inflammatory signaling [90]. This study therefore demonstrates that peptides targeting ubiquitination pathways may inhibit TLR responses.

## Conclusions

Collectively, our data demonstrates the potential of the inhibition of Bid as a co-therapeutic regulator of inflammatory pathways, specifically targeting TLR-induced ubiquitin signaling. Targeting innate immune signaling in the CNS is critical for the attenuation of adaptive immune cell infiltration and a chronic pro-inflammatory response. This study further highlights the immunoregulatory role of Bid and demonstrates a central role of Bid in modulating protein interactions, specifically the A20-mediated deubiquitination of E3 ubiquitin ligases in TLR-induced pro-inflammatory pathways.

## Additional file

Additional file 1: **Figure S1.** *Increased A20-TRAF3*, *A20-Peli1*, *and TRAF6-Peli1 interactions in TLR3- and TLR4-stimulated bid-deficient glia and macrophages, compared with wt.* (A) wt and *bid*^*−/−*^ mixed glia were transfected with Ubiquitin-HA and stimulated for 1 h with LPS (100 ng/ml) 20 h following transfection. Co-immunoprecipitation of anti-TRAF6 was carried out for each sample, and TRAF6-A20 interactions were determined by Western blot using an anti-A20 antibody. TRAF6-A20 interactions were quantified using optical density (*n* = 1 experiment). (B) wt and *bid*-deficient mixed glia were transfected with TRAF6-FLAG and stimulated for 1 h with LPS (100 ng/ml) 20 h post transfection. The cells were lysed in RIPA buffer, and anti-FLAG was immunoprecipitated from each of the samples. Peli1 was detected by Western blot, indicating the interaction between TRAF6-FLAG and Peli1 (*n* = 1 experiment). (C) wt and *bid*-deficient macrophages were stimulated with PolyI:C (100 ng/ml) or LPS (100 ng/ml) for 1 h and lysed in RIPA buffer. Anti-Peli1 was co-immunoprecipitated from each sample, and Peli1-A20 interactions were determined by Western blot using an anti-A20 antibody (*n* = 1 experiment). (TIFF 1770 kb)

## Abbreviations

Bid: BH3-interacting domain death agonist
CNS: Central nervous system
DAMPs: Danger-associated molecular pattern
DUBs: De-ubiquitinating enzymes
IKKε: IҡB kinase ε
IRF3: Interferon regulatory factor 3
JNK: Jun N-terminal kinases
LPS: Lipopolysaccharide
MAPK: Mitogen-activated protein kinase
MyD88: Myeloid differentiation primary response 88
NF-ҠB: Nuclear factor ҠB
NOD: Nucleotide-binding oligomerization domain-containing protein
PAMPs: Pathogen-associated molecular patterns
Peli1: Pellino 1
TAK1: Transforming growth factor β-activated kinase 1
TBK1: Tank-binding kinase 1
TLR: Toll-like receptor
TRAF3/6: TNF receptor associated factor 3/6
TRIF: TIR-domain-containing adapter-inducing interferon-β
