# Supplementary figures and images for: Increased A20-E3 ubiquitin ligase interactions in bid-deficient glia attenuate TLR3- and TLR4-induced inflammation
Source: J Neuroinflammation. 2018 May 2;15:130. doi: 10.1186/s12974-018-1143-3 (PMC5930864; doi:10.1186/s12974-018-1143-3)

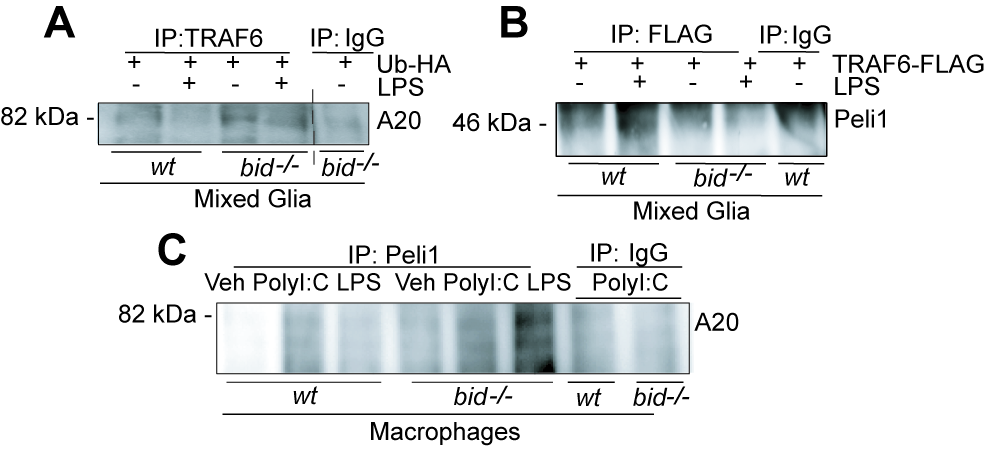

Supplement: Supplementary file 1 — Figure S1. Increased A20-TRAF3, A20-Peli1, and TRAF6-Peli1 interactions in TLR3- and TLR4-stimulated bid-deficient glia and macrophages, compared with wt. (A) wt and bid−/− mixed glia were transfected with Ubiquitin-HA and stimulated for 1 h with LPS (100 ng/ml) 20 h following transfection. Co-immunoprecipitation of anti-TRAF6 was carried out for each sample, and TRAF6-A20 interactions were determined by Western blot using an anti-A20 antibody. TRAF6-A20 interactions were quantified using optical density (n = 1 experiment). (B) wt and bid-deficient mixed glia were transfected with TRAF6-FLAG and stimulated for 1 h with LPS (100 ng/ml) 20 h post transfection. The cells were lysed in RIPA buffer, and anti-FLAG was immunoprecipitated from each of the samples. Peli1 was detected by Western blot, indicating the interaction between TRAF6-FLAG and Peli1 (n = 1 experiment). (C) wt and bid-deficient macrophages were stimulated with PolyI:C (100 ng/ml) or LPS (100 ng/ml) for 1 h and lysed in RIPA buffer. Anti-Peli1 was co-immunoprecipitated from each sample, and Peli1-A20 interactions were determined by Western blot using an anti-A20 antibody (n = 1 experiment). (TIFF 1770 kb) [file 12974_2018_1143_MOESM1_ESM.tif]
